# Evaluation of the dose reduction effect of crystalline lens exposure in cone‐beam computed tomography with bismuth eye shield for image‐guided radiation therapy: An anthropomorphic phantom study

**DOI:** 10.1002/acm2.70024

**Published:** 2025-02-18

**Authors:** Tatsuya Yoshida, Koji Sasaki, Yoshiyuki Kawasaki, Tomoki Hayakawa, Toshiyuki Kawadai, Takako Shibasaki

**Affiliations:** ^1^ Department of Radiology Koritsu Tatebayashi Kosei General Hospital Gunma Japan; ^2^ Graduate School of Radiological Technology Gunma Prefectural College of Health Sciences Gunma Japan; ^3^ Division of Radiation Oncology Nippon Medical School Hospital Tokyo Japan

**Keywords:** crystalline lens, cone‐beam computed tomography, image‐guided radiation therapy, eye shield, phantom movement

## Abstract

This study aimed to evaluate the dose‐exposure reduction effect of a crystalline lens with a bismuth eye shield using cone‐beam computed tomography (CBCT) for head image‐guided radiation therapy. The ocular surface dose of the head phantom (THRA‐1) is defined as a crystalline lens exposure dose and is measured using a radiophotoluminescence dosimeter (RPLD, GD‐352 M) with and without an eye shield (CT eye shield) while moving the head phantom from the reference position that is set at the center of the head in either the *X* or *Z* direction from −5 to +5 cm. The exposure doses were measured thrice at each movement position. The crystalline lens exposure doses at the reference position were 0.896 ± 0.024 mGy and 0.892 ± 0.016 mGy for the right and left sides, respectively. The exposure doses at the position where the head phantom was moved 5 cm in the −*Z* direction from the reference position were 2.812 ± 0.053 mGy and 2.576 ± 0.038 mGy for the right and left sides, respectively, with the highest doses at all movement positions. The crystalline lens exposure doses were reduced to 1.909 ± 0.046 mGy and 1.768 ± 0.043 mGy for the right and left sides with an eye shield in this position, causing an exposure dose reduction rate of −32% and −31%, respectively. The crystalline lens exposure dose reduction rate was approximately 10%–15% in the movement directions, except for the −*Z* direction. Head CBCT with an eye shield effectively reduced the crystalline lens exposure dose when the CBCT isocenter was set close to the eye. Head CBCT using an eye shield is a useful method that reduces the crystalline lens exposure dose.

## 1 INTRODUCTION

The predominant use of high‐precision radiotherapy in modern radiotherapy, such as intensity‐modulated radiation therapy (IMRT) and stereotactic radiotherapy (SRT), reduced the organ at risk dose and created optimized dose distribution for the target.^1^, ^2^, ^3^ In particular, treatment planning for the head and neck radiotherapy aimed to minimize the crystalline lens dose.^4^, ^5^, ^6^ Crystalline lens exposure has been discussed, and the threshold doses for developing cataracts are 1.5 and 8 Gy for acute and chronic exposures, respectively.^7^ However, ICRP Publ. 118 recommended a threshold dose of 0.5 Gy.^8^ Conversely, Nakajima et al.^9^ conducted an epidemiological study that involved 730 atomic bomb survivors and revealed 0.6 and 0.7 Sv threshold dose point estimates for cortical cataracts and posterior subcapsular opacity, respectively. However, the thresholds for cortical cataracts and posterior subcapsular opacities did not exceed 0 Sv, considering that the lower 90% confidence limit was 0 Sv. Furthermore, Neriishi et al.^10^ conducted an epidemiological study that involved 3761 atomic bomb survivors (479 of whom underwent cataract surgery) who underwent biennial health examinations and revealed 0.1 Gy as the best dose threshold estimate (95% confidence interval [CI]: <0–0.8 Gy) after adjusting for age, sex, diabetes mellitus, and other potential confounders. Therefore, a cataract threshold dose of 0.5 Gy is dangerous and the crystalline lens exposure dose should be reduced as much as possible. In contrast, image‐guided radiation therapy (IGRT) is important for safe and accurate high‐precision radiotherapy delivery, but the increased exposure dose has become an issue.^11^, ^12^, ^13^ The cone‐beam computed tomography (CBCT) isocenter during IGRT is frequently the same as the irradiation center, and the position of the CBCT isocenter changes based on the target position, thereby also changing the exposed site. Nelson et al.^14^ demonstrated a 0.15 cGy variation in the crystalline lens exposure dose from CBCT at three CBCT isocenters set 5 cm apart in the anteroposterior direction for a D50 range of 0.03–0.31 cGy. Therefore, the CBCT exposure dose should be managed based on the CBCT isocenter position. The Task Group (TG) 180 report of the American Association of Physicists in Medicine (AAPM)^15^ recommends optimizing the exposure dose while ensuring position‐matching accuracy. The treatment planning process should consider 5% of the therapeutic target dose as the exposure dose threshold, and the as low as reasonably achievable (ALARA) principle should always be applied in practice. In other words, the exposure dose during IGRT should be managed and reduced. Regarding methods to reduce the crystalline lens exposure dose, an eye shield is in clinical use, and its effectiveness has already been reported.^16^, ^17^, ^18^ Kawauchi et al.^18^ reported the effect of eye shields made of different materials, such as lead and bismuth, which reduce the lens exposure dose during angiographic CBCT. However, this study did not consider the CBCT isocenter position movement (phantom movement). To the best of our knowledge, the effect of CBCT isocenter position movement on the crystalline lens exposure dose reduction during CBCT using an eye shield remains unknown.

This study aimed to assess the effect of CBCT isocenter position movement on the crystalline lens exposure dose reduction during head CBCT using an eye shield.

## 2 MATERIALS AND METHODS

### 2.1 Geometric arrangement of measurements

**FIGURE 1:**
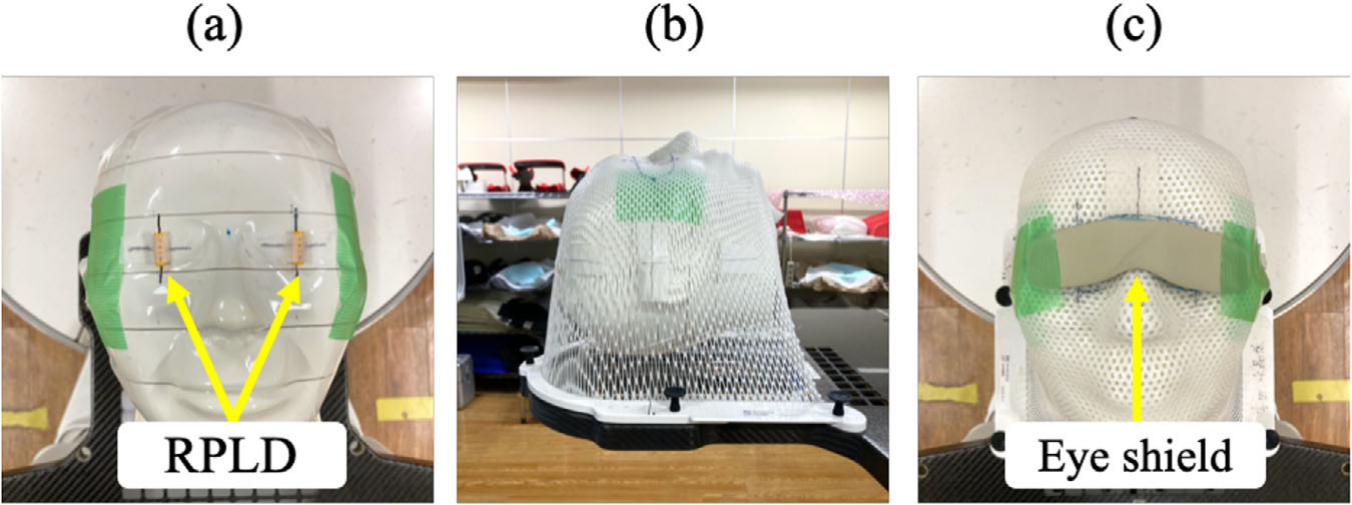
Photographs illustrating geometric arrangements for crystalline lens exposure dose dosimetry. (a) RPLD placed on the ocular surface, (b) head phantom fixed with a shell, and (c) eye shield placed on the shell. RPLD: radiophotoluminescence dosimeter.

We constructed a measurement system based on the method of Yoshida et al. for measuring the crystalline lens exposure dose.^19^ To reproduce the head radiotherapy setup, a head phantom (THRA‐1, Kyoto Kagaku, Kyoto, Japan), was placed on a baseplate (20CFHNSUB2, CIVCO, Orange City, Iowa, USA) and pillow (MTSILVER2B, CIVCO Medical Solutions, Iowa, USA), with the radiophotoluminescence dosimeter (RPLD, GD‐352 M, Chiyoda Technol, Tokyo, Japan) on the right and left eye surfaces of the head phantom (Figure 1a) and fixed with a shell (MTAPUD2.4, CIVCO, Orange City, Iowa) (Figure 1b). The dose measured using the RPLD in this geometric arrangement is defined as the “crystalline lens exposure dose.” An eye shield (CT eye shield, KIRAN, Navi Mumbai, India) was placed over the shell to cover the eyes of the head phantom to reduce the crystalline lens exposure dose (Figure 1c). The eye shield has an eye mask‐like design measuring 133 mm × 33 mm × 0.5 mm, weighing 5 g, and composing a bismuth‐based material with lead equivalent of 0.07 mmPb. The CBCT isocenter was set at the center of the head phantom in the left–right (*X*) and anterior–posterior (*Z*) directions and the superior–inferior (*Y*) direction at the eye position, which was defined as the reference position in the phantom coordinates in IEC61217.^20^

### 2.2 Measurement of crystalline lens exposure dose during CBCT with and without an eye shield

A gantry‐mounted kV X‐ray imaging system (On‐Board Imager: OBI, Varian Medical Systems, Palo Alto, USA) on a linear accelerator (Clinac iX, Varian Medical Systems, Palo Alto, USA) was used to measure the crystalline lens exposure dose. The CBCT imaging conditions were set to Standard‐dose head protocol (100 kV, 145 mAs, 292°–88° counterclockwise (CCW) X‐ray tube rotation angle), and a full‐fan bow‐tie filter attachment (B300882R01C_1252, Varian Medical Systems, Palo Alto, USA). This imaging condition was chosen because we evaluate the crystalline lens exposure dose in a standard CBCT imaging condition. The exposure dose was measured thrice at the reference position and at each position where the head phantom was moved from −5 to +5 cm in either the X or Z direction from the reference position to evaluate changes in the crystalline lens exposure dose depending on the phantom movement. The same measurements were performed to evaluate the effect of the eye shield on reducing crystalline lens exposure doses by moving the head phantom with an eye shield covering the ocular surface of the head phantom.

### 2.3 Calculation of absorbed and exposure dose reduction rates of the crystalline lens

The manufacturer provided RPLDs used in this study after confirming the traceability was assured by the calibration laboratory. The RPLD was annealed, and the background values were read using a glass dosimeter reader (FDG‐1000, Chiyoda Technol, Tokyo, Japan) before the crystalline lens exposure dose measurement. The measured values were obtained by reading the fluorescence level using the glass dosimeter reader after 1 week to sufficiently stabilize the fluorescence of radiophotoluminescence because the RPLD was exposed to the CBCT scan.

In this study, the RPLD was placed on the ocular surface of the head phantom, and values, including backscatter, were measured. Because the crystalline lens is located deeper than the ocular surface, the RPLD must be placed slightly deeper than the surface to accurately measure the crystalline lens exposure dose. The dose measured with the RPLD placed on the ocular surface does not include attenuation due to depth. Therefore, the absorbed dose to the crystalline lens was calculated using the following formula to avoid underestimating the crystalline lens exposure dose:

(1) $$D=\left(M-M_{\mathrm{BG}}\right)\;\times \;\frac{{\left(\frac{\mu_{\mathrm{en}}}{\rho }\right)}_{\mathrm{lens}}}{{\left(\frac{\mu_{\mathrm{en}}}{\rho }\right)}_{\mathrm{air}}}$$

where *D* denotes the dose absorbed by the crystalline lens, *M* represents the measured value, *M*_BG_ indicates the background fluorescence value, and mGy reflects the unit for all measurements. Additionally, ${\left(\frac{\mu_{\mathrm{en}}}{\rho }\right)}_{\mathrm{lens}}$ is the mass‐energy absorption coefficient of the crystalline lens and ${\left(\frac{\mu_{\mathrm{en}}}{\rho }\right)}_{\mathrm{air}}$ is the mass‐energy absorption coefficient of the air. The mass‐energy absorption coefficient was collected from the Seltzer and Hubbell photon attenuation data.^21^ Additionally, Hsu and Kim^22^, ^23^ revealed that GD‐352 M demonstrated good energy characteristics of >30 keV, due to the improved energy dependence of the Sn filter. Trivedi et al.^24^ demonstrated an effective energy of 61 keV for the same CBCT imaging conditions as those used in this study (100 kV, 145 mAs, with a full‐fan bow‐tie filter). Therefore, this study did not consider the energy dependence of GD‐352 M.

The crystalline lens exposure dose reduction rate was calculated using the following formula to evaluate the crystalline lens exposure dose reduction effect based on the phantom movement:

(2) $$\varepsilon =\frac{D_{\mathrm{with}}-D_{\mathrm{without}}}{D_{\mathrm{without}}}\;\times \;100$$

where *D*_with_ denotes the mean value of the absorbed dose to the crystalline lens with an eye shield, *D*_without_ represents the mean value of the absorbed dose to the lens without an eye shield, and mGy denotes the unit for both.

## 3 RESULTS

### 3.1 Crystalline lens exposure dose with and without an eye shield

**FIGURE 2:**
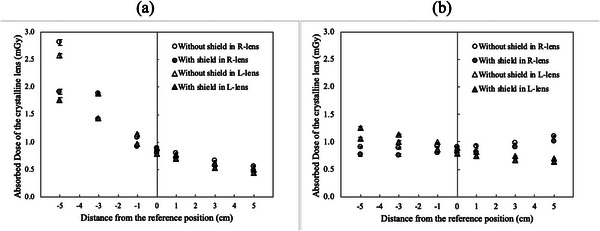
Crystalline lens exposure dose with and without an eye shield. (a) Movement in Z direction and (b) movement in X direction. The ordinate depicts the absorbed dose of the crystalline lens (mGy), and the abscissa represents the amount of phantom movement (cm). The circles indicate the right crystalline lens with (gray) and without (white) an eye shield, the triangles denote the left crystalline lens with (gray) and without (white) an eye shield, and the error bars represent the standard deviation of the three measurements.

Figure 2 illustrates the absorbed dose of the crystalline lens during phantom movement in the X or Z direction with and without an eye shield. Table 1 shows the effect of using an eye shield on reducing the crystalline lens exposure dose. The coefficient of variation (CV) calculated from the results of three measurements of the crystalline lens exposure dose at each movement position was a maximum of 0.035. AAPM TG 12^25^ defines <0.1 as the CV for the X‐ray output of the radiographic units. The CV in this study was much smaller than that of TG 12, even though it includes the uncertainty of the RPLD (sensitivity of the glass element and reading accuracy) and the placing positional accuracy of the RPLD in addition to X‐ray output variation. The absorbed doses to the crystalline lens without an eye shield were 0.896 ± 0.024 mGy and 0.892 ± 0.016 mGy for the right and left sides, respectively, when the head phantom was set at the reference position (Figure 2a and b). The absorbed doses to the crystalline lens at the position where the head phantom was moved 5 cm in the +*Z* direction from the reference position were 0.563 ± 0.005 mGy and 0.492 ± 0.009 mGy for the right and left sides, which increased to 2.812 ± 0.053 mGy and 2.576 ± 0.038 mGy, respectively, when moved 5 cm in the −*Z* direction (Figure 2a). The absorbed dose to the crystalline lens with an eye shield at the position where the head phantom was moved 5 cm in the −*Z* direction decreased to 1.909 ± 0.046 mGy and 1.768 ± 0.043 mGy for the right and left sides (Figure 2a), with the calculated dose reduction rates of −32% and −31%, respectively (Table 1). The dose reduction rates were the highest for all movement positions. The absorbed dose to the right crystalline lens increased to 1.103 ± 0.006 mGy when the head phantom moved 5 cm from the reference position in the +*X* direction, but it did not change the exposure dose when the head phantom moved 5 cm in the −*X* direction (0.902 ± 0.011 mGy) (Figure 2b). The absorbed dose to the left crystalline lens decreased to 0.696 ± 0.015 mGy when the head phantom moved 5 cm from the reference position in the +*X* direction, and it increased to 1.245 ± 0.032 mGy when the head phantom moved 5 cm in the −*X* direction (Figure 2b). The change in the absorbed dose to the crystalline lens was smaller during movement in the *X* direction than in the *Z* direction, with a dose reduction rate with the eye shield of approximately 10%–15% (Table 1).

**TABLE 1:** Crystalline lens exposure dose reduction rate with an eye shield depending on the phantom position.

		Amount of movement (cm)						
Direction of movement	Crystalline lens	−5	−3	−1	0	1	3	5
*Z*	Right	−32	−25	−15	−11	−12	−13	−14
	Left	−31	−24	−16	−12	−10	−11	−12
*X*	Right	−15	−15	−13	−11	−11	−8	−9
	Left	−15	−12	−13	−12	−10	−9	−10

## 4 DISCUSSION

To manage crystalline lens exposure dose in head CBCT, it is important to understand how crystalline lens exposure dose changes depending on the position of the CBCT isocenter. This would help to avoid unexpectedly high exposure. However, there have been no studies on how to reduce the crystalline lens exposure dose, including moving the CBCT isocenter position. The reason is that the change in the crystalline lens exposure dose during CBCT, depending on the isocenter position and the dose reduction effect of shielding, is generally unknown. We have proposed an exposure dose reduction method using the eye shield to effectively reduce the crystalline lens exposure dose depending on the CBCT isocenter position. This is the first study to reduce the crystalline lens exposure dose depending on the CBCT isocenter position.

We converted the absorbed dose in air to the absorbed dose at the ocular surface of a head phantom using Equation (1). In a preliminary study, we measured and compared the absorbed dose with and without a 3‐mm‐thick water‐equivalent bolus placed on the RPLD attached to the ocular surface of a head phantom. In the right eye, the absorbed dose was 0.321 ± 0.018 mGy without a bolus and 0.320 ± 0.009 mGy with the bolus, and in the left eye, the absorbed dose was 0.337 ± 0.016 mGy without the bolus and 0.336 ± 0.011 mGy with the bolus. The dose differences between the doses with and without a bolus were smaller than the variation in the measured values. Therefore, when evaluating the absorbed dose of the crystalline lens from the RPLD measurement, we justified that directly using the result of Equation (1) with reason.

We assessed the changes in the crystalline lens exposure dose with phantom movement during head CBCT and revealed the effect of the eye shield on reducing the crystalline lens exposure dose. The crystalline lens exposure doses in this study were 0.896 ± 0.024 mGy and 0.892 ± 0.016 mGy for the right and left sides, respectively, when the head phantom was set in the reference position. Palomo et al.^26^ reported that the crystalline lens exposure dose in the Standard‐dose head protocol measured with an anthropomorphic phantom and thermoluminescence dosimeters was 0.71 ± 0.07 mGy. This result was similar to our study. Nelson et al.^14^ used CT images of nine patients under the same CBCT imaging conditions as in this study (100 kV, 145 mAs, with full‐fan bow‐tie filter) to calculate the exposure dose from head CBCT using Monte Carlo simulation and revealed 0.3–3.1 mGy crystalline lens exposure dose in the D50 range. Therefore, the crystalline lens exposure dose used in this study was within the range reported by Nelson et al. The crystalline lens exposure doses were 2.812 ± 0.053 mGy and 2.576 ± 0.038 mGy for the right and left sides, when the head phantom was moved 5 cm in the −*Z* direction, and 0.563 ± 0.005 mGy and 0.492 ± 0.009 mGy when moved 5 cm in the +*Z* direction, respectively. The increments in the crystalline lens doses in the *Z* direction compared to the crystalline lens exposure doses at the reference position were 1.916 and 1.684 mGy, and the decrements were 0.333 and 0.4 mGy for the right and left sides, respectively. Regarding the change in crystalline lens exposure dose with phantom movement, Nelson et al. revealed a 1.5 mGy variation in the crystalline lens exposure dose when the isocenter was moved 5 cm. Therefore, the crystalline lens exposure dose in this study is consistent with a previous study.^14^ In addition to this scan protocol, a Low‐dose head protocol (100 kV, 72 mAs) and a High‐quality head protocol (100 kV, 720 mAs) are available on our system. Because the parameters other than mAs are the same in these scan protocols, the mAs ratios to the Standard‐dose head protocol are 0.5 and 5.0, respectively. Therefore, the crystalline lens exposure dose in the other two conditions could be calculated numerically. On the other hand, regarding techniques to reduce the crystalline lens exposure dose, Ding et al.^27^ reported a Monte Carlo study comparing the crystalline lens exposure dose when the X‐ray tube rotation angle for CBCT of the head was rotated 360° and 200° below and above the patient. First, Ding et al. demonstrated that the maximum dose to the eyes at the same X‐ray tube rotation angle below the patient (290°–90°) as in our study was approximately 2.5 mGy, which is comparable to the crystalline lens exposure dose in our study. Additionally, Ding et al. exhibited that the dose reduction for the eyes was approximately 70% at a 200° X‐ray tube rotation angle with the X‐ray tube rotating below the patient compared to a 200° angle with the X‐ray tube rotating above the patient. This X‐ray tube rotation angle significantly cuts the primary X‐ray to the eye, thereby producing a high dose reduction effect. This X‐ray tube rotation angle further reduce the crystalline lens exposure dose by a maximum of approximately 30% using the eye shield. The crystalline lens exposure dose should be as low as possible; thus, using an eye shield to reduce the exposure dose is significant to minimize the exposure risk.

**FIGURE 3:**
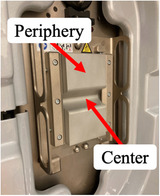
Full‐fan bow‐tie filter. The center is thinner than the periphery.

We discuss the changes in the crystalline lens exposure dose with phantom movement and the effect of eye shields on the exposure dose reduction. The cases with increased absorbed doses by the phantom movement were the left and right in the −*Z* direction, the right in the +*X* direction, and the left in the −*X* direction. Blessing et al.^28^ revealed that the dose profile in the air of a full‐fan bow‐tie filter calculated using Monte Carlo simulation varied steeply with the full‐fan bow‐tie filter thickness within a ±10‐cm distance from the isocenter (1.0–0.2 relative range with the image center dose of 1.0). The CBCT isocenter was exposed to high‐dose X‐rays that passed through the center compared to those passing through the periphery of the full‐fan bow‐tie filter (Figure 3). Therefore, the crystalline lens exposure dose increased as the phantom moved toward the CBCT isocenter. This increase when moved in the −*Z* direction compared to the X direction, may be due to the change in the positional relationship between the crystalline lens and the surrounding bone in terms of the view from the X‐ray tube, increased the number of views in which the crystalline lens was not covered by bone, thereby increasing primary X‐rays incident on the crystalline lens. Therefore, the increased factors of the crystalline lens exposure dose when the crystalline lens moves to the CBCT isocenter include the increase in the incident dose, which depends on the shape of the full‐fan bow‐tie filter, and the increase in primary X‐rays to the crystalline lens because of the change in the positional relationship between the crystalline lens and bone in terms of the X‐ray tube caused by phantom movement. A movement of 5 cm in the −*Z* direction, during which the crystalline lens exposure dose notably increased, demonstrated the greatest dose reduction effect (the dose reduction of approximately 30%) because the eye shield effectively shielded the X‐rays incident on the crystalline lens. However, the dose reduction rate of the crystalline lens by the eye shield in a CT shown in the manufacturer's literature is 70%; thus, the dose reduction effect in this study was lower than that in the manufacturer's literature. In the CBCT with an X‐ray tube rotation angle of 360°, more primary X‐rays are incident on the crystalline lens than the 200° rotation angle in this study. The higher dose reduction effect than in this study is due to the effective shielding of primary X‐rays to the crystalline lens by the eye shield. The factor changing in the crystalline lens exposure dose and dose reduction effect with the phantom position change includes the dose of primary X‐rays, which changes depending on the shape of the bow‐tie filter. Therefore, we believe that changes in the crystalline lens exposure dose with the movement of the patient position in this study will be similar to other CBCT systems and imaging protocols using bow‐tie filters. Even if the anatomy and physique of the patient differ, the change in the crystalline lens exposure dose and dose reduction effect should be similar because the incident dose to the crystalline lens changes consistently with the change in the patient's position.

We discuss the clinical use of the eye shield. The eye shield can be used universally for head CBCT because it reduces the crystalline lens exposure dose regardless of the CBCT isocenter position. The dose reduction effect is higher when the CBCT isocenter is located anterior of the head; thus, the eye shield effectively reduces the crystalline lens exposure dose during radiotherapy for tumors in the frontal and temporal lobes. On the other hand, several additional clinical workflows must be required when the eye shield is actually used in clinical practice. Initially, the additional workflow is placing the eye shield over the eye after fixing the head with a shell. This flow is similar to placing the bolus material over the skin and could easily be added. However, clinical practice must consider the possibility of forgetting to place the eye shield. Therefore, we recommend adding a flow to confirm the setup using an easily accepted bar code or similar. Next, scattered radiation may result when the treatment beam is irradiated onto the eye shield; therefore, a process is required to remove the eye shield before irradiation. This process can be added or not by confirming with the beam's eye view that the structure simulating the eye shield created during treatment planning is not irradiated. We recommend adding a flow when adding a flow to remove the eye shield to confirm that the eye shield has been securely removed (e.g., by double‐checking or using a check sheet). These additional flows should be considered to reduce the crystalline lens exposure dose safely.

**FIGURE 4:**
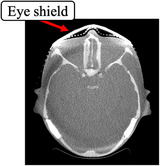
Transverse image of a head phantom CBCT with an eye shield. CBCT: cone‐beam computed tomography.

Images obtained using an eye shield have metal artifacts where the eye shield contacts the skin (Figure 4). Fricke et al.^29^ proposed a method in which a 1‐cm space is maintained between the eye shield and body surface (e.g., by inserting a sponge) to improve the image quality of CT images. This method should improve image quality in CBCT with the eye shield. Additionally, Kawauchi et al.^18^ reported that the dose reduction effect for the crystalline lens in a split eye shield to cover only the eye and an unsplit eye shield was the same, with reduced artifacts from the eye shield. Specifically, the eye shield with a smaller and thicker design covering only the eye and a 1‐cm thickness sponge between the eye shield and the shell surface may further reduce the crystalline lens exposure dose while minimizing image degradation.

Herein, we discuss the crystalline lens exposure dose in clinical cases of head radiotherapy. The number of CBCT exposures during the treatment period differs among treatment institutes. Still, the fact that the crystalline lens exposure dose in head CBCT changes significantly depending on the number of CBCT exposures is consistent. Therefore, treatment institutes must manage the crystalline lens exposure dose according to the number of CBCT exposures. Additionally, the addition of the eye shield could reduce the incremental crystalline lens exposure dose. In clinical cases, maximum crystalline lens exposure doses without an eye shield in this study were 2.812 and 2.576 mGy for the right and left sides, respectively. In the SRT for 21–27 Gy per three fractions,^30^ the calculating crystalline lens exposure doses with one CBCT per fraction are 8.436 and 7.728 mGy for the right and left sides, respectively. In the radiotherapy for 60 Gy glioblastoma per 30 fractions,^31^ the calculating crystalline lens exposure doses with one CBCT per fraction are 84.36 and 77.28 mGy for the right and left sides, respectively. Since the crystalline lens exposure dose varies significantly depending on the number of fractions, managing it as described above is very important.

This study demonstrated the substantially increased crystalline lens exposure dose when the head phantom was moved 5 cm in the −*Z* direction. However, the cause of this increase has not been investigated in detail. Future research is warranted to investigate the factors that increased the incident dose to the crystalline lens when the head is moved to the CBCT isocenter. Additionally, we would like to study the dose reduction effect for the crystalline lens and the evaluation of the image quality with a smaller and thickened eye shield to improve the dose reduction effect and reduce artifacts.

## 5 CONCLUSION

The crystalline lens exposure dose with phantom movement was measured during head CBCT, and the effect of the eye shield on dose reduction was demonstrated. To the best of our knowledge, no reports have evaluated the increase in the crystalline lens exposure dose according to changes in the phantom position, which further evaluates the enhanced exposure dose reduction effect of the eye shield in positions where crystalline lens exposure dose increases. The crystalline lens exposure dose notably increased as the phantom position moved in the −*Z* direction (when the CBCT isocenter was set close to the eye). Additionally, the largest dose reduction of approximately 30% was achieved when the eye shield was used at this position. Furthermore, the exposure dose reduction effect of the eye shield was observed in the *X* and +*Z* directions (approximately 10%–15%). Head CBCT, using an eye shield, is a useful method for reducing the crystalline lens exposure dose.

## AUTHOR CONTRIBUTIONS

Planned the study: Tatsuya Yoshida. Measured and analyzed the data: Tatsuya Yoshida, Tomoki Hayakawa, Toshiyuki Kawadai, Takako Shibasaki. Supervised the study: Koji Sasaki, Yoshiyuki Kawasaki. Wrote the manuscript: Tatsuya Yoshida, Koji Sasaki. All authors contributed to the review of the manuscript and all approved the final draft for submission.

## CONFLICT OF INTEREST STATEMENT

The authors declare no conflicts of interest.

## ETHICS STATEMENT

This study did not involve human subjects as such ethical approval was not required.

## Data Availability

Data sharing does not apply to this article as no datasets were generated or analyzed during the current study.
